# Male circumcision and prevalence of genital human papillomavirus infection in men: a multinational study

**DOI:** 10.1186/1471-2334-13-18

**Published:** 2013-01-17

**Authors:** Ginesa Albero, Luisa L Villa, Eduardo Lazcano-Ponce, William Fulp, Mary R Papenfuss, Alan G Nyitray, Beibei Lu, Xavier Castellsagué, Martha Abrahamsen, Danélle Smith, F Xavier Bosch, Jorge Salmerón, Manuel Quiterio, Anna R Giuliano

**Affiliations:** 1 Unit of Infections and Cancer (UNIC), Cancer Epidemiology Research Program (CERP), Catalan Institute of Oncology (ICO), L'Hospitalet de Llobregat 08908, Barcelona, Spain; 2 CIBER en Epidemiología y Salud Pública – CIBERESP (Epidemiology and Public Health Biomedical Research Consortium), Barcelona, Spain; 3 Program in Public Health and the Methodology of Biomedical Research, Universitat Autonoma de Barcelona (UAB). Campus Universitat Autonoma, s/n, Cerdanyola del Valles, 08193, Barcelona, Spain; 4 Ludwig Institute for Cancer Research, São Paulo, 01323-903, Brazil; 5 Instituto Nacional de Salud Publica, Cuernavaca, 62100, Mexico; 6 H. Lee Moffitt Cancer Center and Research Institute, Tampa, FL, 33612, USA; 7 Instituto Mexicano del Seguro Social, Cuernavaca, 62140, Mexico

**Keywords:** Male circumcision, Genital, HPV, Non-oncogenic, Prevalence

## Abstract

**Background:**

Accumulated evidence from epidemiological studies and more recently from randomized controlled trials suggests that male circumcision (MC) may substantially protect against genital HPV infection in men. The purpose of this study was to assess the association between MC and genital HPV infection in men in a large multinational study.

**Methods:**

A total of 4072 healthy men ages 18–70 years were enrolled in a study conducted in Brazil, Mexico, and the United States. Enrollment samples combining exfoliated cells from the coronal sulcus, glans penis, shaft, and scrotum were analyzed for the presence and genotyping of HPV DNA by PCR and linear array methods. Prevalence ratios (PR) were used to estimate associations between MC and HPV detection adjusting for potential confounders.

**Results:**

MC was not associated with overall prevalence of any HPV, oncogenic HPV types or unclassified HPV types. However, MC was negatively associated with non-oncogenic HPV infections (PR 0.85, 95% confident interval: 0.76-0.95), in particular for HPV types 11, 40, 61, 71, and 81. HPV 16, 51, 62, and 84 were the most frequently identified genotypes regardless of MC status.

**Conclusions:**

This study shows no overall association between MC and genital HPV infections in men, except for certain non-oncogenic HPV types for which a weak association was found. However, the lack of association with MC might be due to the lack of anatomic site specific HPV data, for example the glans penis, the area expected to be most likely protected by MC.

## Background

Genital human papillomavirus (HPV) infection is one of the most common sexually transmitted infections (STI) in the world [1]. Genital HPV prevalence in men has been reported to range from 2% to 93% among high-risk men such as HIV-positive males, male partners of women with HPV infection or abnormal cytology, and patients attending STI clinics and from 1% to 84% among all other groups [2]. Most observational studies have shown an inverse association between male circumcision (MC) and genital HPV infection [3-9]. Three randomized controlled trials (RCT) in Africa have shown that MC substantially reduces HIV infection in men [10-12], and two of these have also found that MC reduced the prevalence of high risk HPV infections [13,14]. A recent systematic review and meta-analysis showed an inverse association between MC and genital HPV prevalence in men [15]. Another review reported a strong protective effect of MC against penile cancer [16]. However, the association reported between MC and genital HPV infection has not been consistent across all studies [17-21]. We previously published interim data in support of an inverse association between MC and genital HPV detection in the first 1158 men enrolled in the HPV in Men study (*HIM Study*) [6]. In the present analysis, we re-evaluated the association between MC and HPV detection, type distribution and possible effect modification by sexual behavior among all men enrolled in the *HIM Study*.

## Methods

### Study population

The *HIM Study* is a multinational longitudinal study of HPV in men. Between June 2005 and September 2009, 4072 men who completed both the pre-enrollment and the enrollment visits formed the study population for the present analysis. Details of the cohort have been previously described [6,22]. In brief, men were eligible for participation if they were aged 18–70 years, residents of Tampa, Florida, Cuernavaca, Mexico, or São Paulo, Brazil, had no prior anal or penile cancer or genital warts, and no current diagnosis of STIs. Men were recruited from several population sources. In Brazil, men were recruited from the general population, public health clinic attendees, and partners of women participating in a natural history study of HPV. Men visiting the clinic for STI symptoms or treatment were excluded. In Mexico, men were recruited through a large health plan, from factories, and the military. In the United States, men were recruited from the University of South Florida and the Tampa metropolitan area. Men who were eligible to participate reviewed a written informed consent with a trained study team member. Consenting partici-pants underwent a clinical examination at 10 visits: a pre-enrollment visit, an enrollment visit approximately 2 weeks later, and 8 additional visits after enrollment that occurred every 6 months over a period of 4 years. Before study initiation, the human subjects committees of the University of South Florida, the Centro de Referencia e Tratamento de Doencas Sexualmente Transmissiveis e AIDS, Brazil, and the National Institute of Public Health of Mexico approved the research protocol.

At the enrollment visit, men completed an 88-item Computer-Assisted Self-Interview (CASI) that has been demonstrated to elicit reliable responses [23]. The CASI collected information about participant socio-demographic characteristics, tobacco consumption, and sexual behaviors. After the interview, a clinician examined each participant for signs of STIs and assessed circumcision status. Participants with full or partial circumcision were considered to be circumcised.

### Penile and scrotal sampling

Three different saline pre-wetted Dacron (Digene, Gaithersburg, MD, USA) swabs were obtained from the coronal sulcus/glans penis, penile shaft, and scrotum, and placed in 450 μl of Specimen Transportation Medium, and combined into one sample before DNA extraction. Among uncircumcised men, the foreskin was sampled at the time of collection of the coronal sulcus/glans penis sample. We have previously shown the validity and high sampling reproducibility of these three anatomical sites in the assessment of HPV DNA status [24,25]. All HPV samples were stored at −80°C until PCR analysis and genotyping were performed.

### HPV analyses

DNA was extracted with the Media Kit (QIAGen, Valencia,CA, USA) by a robotic system according to the manufacturer’s instructions. DNA was stored at 4°C until use. HPV testing of the combined DNA extract was undertaken by use of PCR for amplification of a fragment of the HPV L1 gene. Specimens were tested for the presence of HPV by amplification of 30 ng of DNA with the PGMY09/11 L1 consensus primer system [26]. HPV genotyping was done with the linear array method on all samples irrespective of the HPV PCR result (Roche Molecular Diagnostics, Alameda, CA, USA) [27].

Before genotyping, amplification products were run on 2% agarose gels to visualise a 450 bp band corresponding to HPV amplification for identification of samples that might have an HPV type other than the 37 types analyzed in the genotyping assay. Samples in which HPV was amplified on PCR, but did not hybridise with a specific HPV type during genotyping were categorized as unclassified infections. The 13 HPV types that were classified as oncogenic were: 16, 18, 31, 33, 35, 39, 45, 51, 52, 56, 58, 59 and 68. The non-oncogenic HPV types were: 6, 11, 26, 40, 42, 53, 54, 55, 61, 62, 64, 66, 67, 69, 70, 71, 72, 73, 81, 82, 83, 84, IS39, and CP6108 [28]. Samples were considered valid if they were HPV positive by PCR or by genotyping, or β-globin positive, regardless of HPV result. Valid samples were available from 3969 of 4072 participants.

### Statistical analysis

The present analysis included four classifications of HPV detection. A participant was considered positive for “Any HPV type” if he tested HPV positive by PCR or by genotyping for any HPV genotype. The category of “Oncogenic HPV” included men who were positive for only oncogenic genotypes as well as those who were positive for both oncogenic and non-oncogenic types. The “Non-oncogenic HPV” category consisted of those participants who tested positive for non-oncogenic HPV genotypes only. The “Unclassified HPV infections” included men with samples that tested positive for HPV by PCR but negative for all of the 37 genotypes tested.

Differences in the distribution of socio-demographic characteristics, tobacco consumption and sexual behavior characteristics by circumcision status were compared using Pearson’s chi-square test. Prevalence ratios (PRs) and 95% confidence intervals (CIs) were estimated using univariate and multivariable modified Poisson regression with robust variance [29,30]. Separate models were constructed for each HPV outcome (i.e., any HPV, oncogenic HPV, non-oncogenic HPV, and unclassified HPV infections). Uncircumcised men served as the reference group for circumcision status, and men who had no detectable HPV (HPV negative on both PCR and genotyping assays) served as the comparison group in the modeling of each HPV outcome. All risk factor variables and potential confounders were initially considered using multivariable modified Poisson regression modeling. For variable selection, all variables in a model were included, and then individually eliminated if not statistically significant at the 0.05 level. This variable selection procedure was performed for the four HPV outcomes. Variables that were not statistically significant in any of the models were excluded from the final multivariable models.

To explore whether sexual behavior modified the association between MC and HPV detection, we stratified the analysis by lifetime number of female sexual partners, and formally tested the interaction between lifetime number of female sexual partners and MC by including in the model both the interaction and the main-effect terms. Statistical analyses were conducted using SAS software, version 9.2 (SAS Institute Inc., Cary, NC) and R 2.13.0 (R Development Core Team). All statistical tests were two sided with a significance threshold of 0.05.

## Results

Socio-demographic characteristics of the 3969 participants by circumcision status are summarized in Table 1. The majority of men were uncircumcised (64.1%). The mean age of participants was 30.9 years among circumcised men and 33.4 years among uncircumcised men. Most circumcised men were recruited from the United States (72.1%) and among uncircumcised men, 46.8% were recruited from Brazil, and 43.8% from Mexico. Among circumcised men, 66.2% were positive for any HPV, 29.4% for oncogenic HPV, 19.7% for non-oncogenic HPV only, and 17.1% for unclassified HPV infections. Among uncircumcised men, 67.0% were positive for any HPV, 30.0% for oncogenic HPV, 24.3% for non-oncogenic HPV, and 12.7% for unclassified HPV infections. Statistically significant differences by circumcision status were observed for age, country of residence, race, marital status, education, sexual orientation, number of female sexual partners in the past 3–6 months, male anal sexual partners in the past 3 months, self-reported STI history, and HPV status.

**Table 1:** Socio-demographic characteristics of study participants by circumcision status N = 3969

	**Circumcision**				**P-value**
	**No**		**Yes**		
	**N = 2545 (64.1%)**		**N = 1424 (35.9%)**		
	**N**	**%**	**N**	**%**	
**Age**					**< 0.001**
Mean (SD)	33.4 (10.3)		30.9 (12.1)		
18-30	1115	43.8	822	57.7	
31-44	1119	44.0	406	28.5	
45-70	311	12.2	196	13.8	
**Country of residence**					**< 0.001**
US	241	9.5	1027	72.1	
Brazil	1190	46.8	197	13.8	
Mexico	1114	43.8	200	14.0	
**Race**					**< 0.001**
White	877	35.1	889	63.1	
Black	397	15.9	218	15.5	
Asian/Pacific Islander	65	2.6	46	3.3	
Mexican	1020	40.8	183	13.0	
Other (American Indian, Mixed)	141	5.6	73	5.2	
Unknown	45		15		
**Marital status**					**< 0.001**
Single	946	37.3	838	58.9	
Married	1035	40.8	322	12.7	
Cohabiting	373	14.7	104	4.1	
Divorced/Separated/Widowed	183	7.2	158	11.1	
Unknown	8		2		
**Education**					**< 0.001**
<12 Years	750	29.6	133	9.3	
Completed 12 Years	774	30.6	292	20.5	
13-15 Years	377	14.9	623	43.8	
Completed 16 Years	497	19.6	273	19.2	
≥17 Years	135	5.3	102	7.2	
Unknown	12		1		
**Current smoker**					0.054
No	1916	75.4	1112	78.2	
Yes	624	24.6	310	21.8	
Unknown	5		2		
**Sexual orientation**					**< 0.001**
MSW	2092	82.5	1245	87.7	
MSWM	161	6.4	54	3.8	
MSM	132	5.2	41	2.9	
No sex	150	5.9	79	5.6	
Unknown	10		5		
**Lifetime female sexual partners**					0.059
Median (SD)	6 (2055.1)		7 (79.4)		
0-1	440	18.5	253	18.4	
2-9	1040	43.8	560	40.8	
10-19	416	17.5	234	17.0	
20-49	354	14.9	229	16.7	
50+	124	5.2	98	7.1	
Unknown	171		50		
**Female sexual partners in past 3–6 months**					**< 0.001**
Mean (SD)	1.4 (2.4)		1.4 (1.7)		
0	717	30.7	329	24.3	
1	940	40.3	668	49.3	
2	349	15.0	163	12.0	
3+	326	14.0	196	14.5	
Unknown	213		68		
**Male anal sexual partners in past 3 months**					**0.002**
Mean (SD)	0.3 (3.3)		0.1 (1.0)		
0	2341	92.9	1363	95.7	
1	75	3.0	25	1.8	
2	31	1.2	16	1.1	
3+	73	2.9	20	1.4	
Unknown	25		0		
**Diagnosis of STIs, ever**					**< 0.001**
No	1992	81.3	1209	86.9	
Yes	459	18.7	183	13.1	
Unknown	94		32		
**HPV status**					**< 0.001**
Negative	840	33.0	481	33.8	
Oncogenic HPV	763	30.0	419	29.4	
Non-oncogenic HPV	618	24.3	280	19.7	
Unclassified HPV	324	12.7	244	17.1	
**HPV multiple infections**					0.169
2+ HPV types	794	57.5	379	54.2	
1 HPV type	587	42.5	320	45.8	
Negative	840		481		

**NOTE. ***SD*, standard deviation; *US*, the United States; *MSW*, men who have sex with women; *MSWM*, men who have sex with women and men; *MSM*, men who have sex with men; *STI*, sexually transmitted infections. Numbers in bold correspond to P values < 0.05.

The univariate and multivariable associations of MC with each of the four pre-defined individual outcomes (i.e., any HPV, oncogenic HPV, non-oncogenic HPV, and unclassified HPV infections) are presented in Table 2. No association was observed between MC and HPV detection for any HPV or oncogenic HPV in either the univariate or the multivariable analyses. However, MC was significantly negatively associated with non-oncogenic HPV detection in both the univariate (PR 0.87, 95% CI 0.78-0.97) and the multivariable models (PR 0.85, 95% CI 0.76-0.95). MC was significantly positively associated with prevalence of unclassified HPV infections in the univariate analysis (PR 1.21, 95% CI 1.05-1.39) but not in the final multivariable analysis (PR 1.07, 95% CI 0.92-1.24).

**Table 2:** Association between male circumcision and genital HPV detection in men

	**Any HPV (n = 3969)**		**Oncogenic HPV (n = 2503)**		**Non-Oncogenic HPV (n = 2219)**		**Unclassified HPV (n = 1889)**	
	**Univariate**	**Multivariable**^**1**^	**Univariate**	**Multivariable**^**1**^	**Univariate**	**Multivariable**^**1**^	**Univariate**	**Multivariable**^**1**^
	**PR (95% CI)**	**PR (95% CI)**	**PR (95% CI)**	**PR (95% CI)**	**PR (95% CI)**	**PR (95% CI)**	**PR (95% CI)**	**PR (95% CI)**
**Circumcision**								
**No**	1.0	1.0	1.0	1.0	1.0	1.0	1.0	1.0
**Yes**	0.99 (0.94 - 1.04)	0.96 (0.91 - 1.01)	0.98 (0.90 - 1.07)	0.95 (0.87 - 1.03)	**0.87 (0.78 - 0.97)**	**0.85 (0.76 - 0.95)**	**1.21 (1.05 – 1.39)**	1.07 (0.92 – 1.24)

**NOTE.***PR*, prevalence ratio; *CI*, confidence interval. Numbers in bold correspond to statistically significant point estimates.

^1^Adjusted for race, marital status, lifetime female sexual partners, female sexual partners in past 3–6 months and male anal sexual partners in the past 3 months.

As shown in Table 3, the association between MC and non-oncogenic HPV differed significantly by the number of lifetime female sexual partners (test of interaction, P = 0.006). Thus, MC was associated with a reduced prevalence of non-oncogenic HPV infections among men reporting 1 to 4 lifetime female sexual partners in both models (univariate PR 0.62, 95% CI 0.45-0.83; multivariable PR 0.59, 95% CI 0.42-0.83). In contrast, no association with MC was observed among men reporting 5 or more lifetime female sexual partners for non-oncogenic HPV. Although in the univariate analysis MC was associated with a statistically significant increased risk of unclassified HPV infections (PR 1.24, 95% CI 1.01-1.51) among men reporting 1–4 partners, the PR was reduced to 1.03 (95% CI 0.82-1.28) in the final multivariable analysis. No associations with MC were observed among men reporting 5 or more lifetime female sexual partners for any HPV, oncogenic HPV, or unclassified HPV infections. The test of interaction between MC and lifetime female sexual partners was not statistically significant for the other three outcomes (i.e., any HPV type, oncogenic HPV, and unclassified HPV infections). Moreover, no association was found between MC and detection of HPV by country of residence (data not shown).

**Table 3:** Association between MC and genital HPV detection in men by lifetime number of female partners

		**Circumcision**		
	**N**	**No**	**Yes**	
			**Univariate**	**Multivariable**^**1**^
			**PR (95% CI)**	**PR (95% CI)**
**Any HPV**				
** Lifetime number of female partners**				P = 0.146^2^
** 1-4**	1099	1.00	0.97 (0.87-1.08)	0.92 (0.81-1.03)
** 5-12**	1183	1.00	0.98 (0.91-1.07)	0.96 (0.88-1.05)
** 13+**	1083	1.00	1.03 (0.97-1.10)	1.03 (0.96-1.10)
**Oncogenic HPV**				
** Lifetime number of female partners**				P = 0.204^2^
** 1-4**	667	1.00	0.93 (0.73 - 1.19)	0.96 (0.73 - 1.26)
** 5-12**	726	1.00	0.96 (0.82 - 1.12)	0.92 (0.77 - 1.09)
** 13+**	697	1.00	1.06 (0.95 - 1.18)	1.06 (0.95 - 1.19)
**Non-Oncogenic HPV**				
** Lifetime number of female partners**				**P = 0.006**^**2**^
** 1-4**	647	1.00	**0.62 (0.45 - 0.83)**	**0.59 (0.42 - 0.83)**
** 5-12**	668	1.00	0.89 (0.74 - 1.07)	0.88 (0.72 - 1.08)
** 13+**	542	1.00	1.03 (0.88 - 1.20)	1.03 (0.88 - 1.21)
**Unclassified HPV**				
** Lifetime number of female partners**				P = 0.952^2^
** 1-4**	717	1.00	**1.24 (1.01 – 1.51)**	1.03 (0.82 – 1.28)^3^
** 5-12**	527	1.00	1.25 (0.96 – 1.61)	1.15 (0.86 – 1.53)^3^
** 13+**	330	1.00	1.19 (0.83 – 1.71)	1.10 (0.74 – 1.62)^3^

**NOTE.***PR*, prevalence ratio; *CI*, confidence interval. Numbers in bold correspond to statistically significant point estimates.

^1^Adjusted for race, marital status, female sexual partners in past 3–6 months and male anal sexual partners in the past 3 months.

^2^Test for interaction between MC and lifetime number of female partners.

^3^Adjusted for race, marital status, and male anal sexual partners in the past 3 months.

Table 4 presents the type-specific HPV distribution by circumcision status. HPV 16, 51, 62, 84, and CP6108 were the most frequently identified genotypes regardless of MC status. Concerning individual HPV genotypes, MC was associated with significantly reduced risk of non-oncogenic HPV types 11 (PR 0.46, 95% CI 0.24-0.88), 40 (PR 0.30, 95% CI 0.15-0.61), 61 (PR 0.50, 95% CI 0.36-0.71), 71 (PR 0.17, 95% CI 0.07-0.43), and 81 (PR 0.55, 95% CI 0.36-0.82). MC was not associated with prevalence of any of the individual thirteen oncogenic HPV types detected.

**Table 4:** Genital type-specific HPV distribution in men by circumcision status

**HPV**	**Circumcision**				**N total**	**PR (95% CI)**
	**No**		**Yes**			
	**N**	**%**	**N**	**%**		
**Oncogenic**						
**16**	190	7.5	117	8.2	307	1.10 (0.88-1.37)
**18**	51	2.0	40	2.8	91	1.40 (0.93-2.11)
**31**	51	2.0	20	1.4	71	0.70 (0.42-1.17)
**33**	20	0.8	4	0.3	24	0.36 (0.12-1.04)
**35**	41	1.6	24	1.7	65	1.05 (0.63-1.72)
**39**	96	3.8	49	3.4	145	0.91 (0.65-1.28)
**45**	47	1.8	18	1.3	65	0.68 (0.40-1.17)
**51**	158	6.2	95	6.7	253	1.07 (0.84-1.37)
**52**	166	6.5	78	5.5	244	0.84 (0.65-1.09)
**56**	55	2.2	20	1.4	75	0.65 (0.39-1.08)
**58**	71	2.8	27	1.9	98	0.68 (0.44-1.05)
**59**	131	5.1	91	6.4	222	1.24 (0.96-1.61)
**68**	54	2.1	37	2.6	91	1.22 (0.81-1.85)
**Non-oncogenic**						
**6**	156	6.1	84	5.9	240	0.96 (0.74-1.24)
**11**	43	1.7	11	0.8	54	**0.46 (0.24-0.88)**
**26**	4	0.2	4	0.3	8	1.79 (0.45-7.14)
**40**	54	2.1	9	0.6	63	**0.30 (0.15-0.60)**
**42**	40	1.6	15	1.1	55	0.67 (0.37-1.21)
**53**	141	5.5	86	6.0	227	1.09 (0.84-1.41)
**54**	56	2.2	34	2.4	90	1.09 (0.71-1.65)
**55**	64	2.5	35	2.5	99	0.98 (0.65-1.47)
**61**	147	5.8	39	2.7	186	**0.47 (0.34-0.67)**
**62**	218	8.6	102	7.2	320	0.84 (0.67-1.05)
**64**	3	0.1	1	0.1	4	0.60 (0.06-5.72)
**66**	124	4.9	81	5.7	205	1.17 (0.89-1.53)
**67**	16	0.6	4	0.3	20	0.45 (0.15-1.33)
**69**	7	0.3	1	0.1	8	0.26 (0.03-2.07)
**70**	71	2.8	29	2.0	100	0.73 (0.48-1.12)
**71**	53	2.1	5	0.4	58	**0.17 (0.07-0.42)**
**72**	39	1.5	12	0.8	51	0.55 (0.29-1.05)
**73**	51	2.0	22	1.5	73	0.77 (0.47-1.27)
**81**	94	3.7	28	2.0	122	**0.53 (0.35-0.81)**
**82**	18	0.7	12	0.8	30	1.19 (0.58-2.47)
**83**	81	3.2	31	2.2	112	0.68 (0.45-1.03)
**84**	197	7.7	119	8.4	316	1.08 (0.87-1.34)
**IS39**	20	0.8	6	0.4	26	0.54 (0.22-1.33)
**CP6108**	162	6.4	113	7.9	275	1.25 (0.99-1.57)

**NOTE.***PR*, prevalence ratio; *CI*, confidence interval. Numbers in bold correspond to statistically significant point estimates.

## Discussion

Our study found that MC was not associated with overall prevalence of genital HPV for any HPV, oncogenic HPV infections, or unclassified HPV infections. However, MC was negatively associated with non-oncogenic HPV infections. After adjustment for potential confounders and stratification by lifetime number of female sexual partners, the protective association between MC and non-oncogenic HPV detection was particularly evident in men reporting 1 to 4 lifetime female sexual partners. The lack of an effect of MC among men with a greater number of lifetime sexual partners might be explained by immunity to HPV acquired over time by men who were repeatedly exposed to HPV infections [6]. Other studies have observed a plateau in risk for HPV detection despite increasing number of lifetime sexual partners [3,6]. However, in another analysis of the *HIM Study,* anti-HPV 16 serum antibody status at enrollment was not associated with the risk of a subsequent genital HPV 16 infection [31].

A preliminary analysis of the HIM study that included the first 1158 men who completed both the pre-enrollment and the enrollment visits, showed that MC was negatively associated with the detection of both non-oncogenic and oncogenic HPV types [6]. However, after increasing the sample size to 3969 men in this analysis, the association was only found for non-oncogenic HPV types after adjusting for potential confounders. Three other studies have reported statistically significant associations between MC and detection of non-oncogenic HPV types [5,14,32]. One MC RCT conducted in South Africa observed significantly lower prevalence of both oncogenic and non-oncogenic HPV types in urethral swab samples among circumcised men than uncircumcised men [13,32]. In another clinical trial in Uganda, the prevalence of both oncogenic and non-oncogenic HPV types using samples of the coronal sulcus/glans penis was lower among circumcised men than uncircumcised men [14]. Further, a study conducted among men attending a public STI clinic, found that MC was associated with reduced risk for both oncogenic and non-oncogenic HPV types using samples of the coronal sulcus/glans penis [5]. However, results from three other studies did not support a protective role of MC on genital non-oncogenic HPV infection [3,19,20].

The study of the effects of MC on genital HPV infections poses inherent complexities. Inconsistency of results across studies may be due to the genital site selected for HPV sampling or by the procedure used to collect exfoliated cells. Most studies sampled only a single anatomical site or a pooled combination of a few sites for the detection of HPV. It has been suggested that sampling of the penile shaft, the coronal sulcus/glans penis, and the scrotum is necessary for optimal overall genital HPV detection [25]. In a US study, an inverse association was found between MC and HPV detection in samples from the urethra, coronal sulcus/glans penis, and penile shaft, but not in samples from the scrotum, semen, anal canal, and perianal area [33]. In another US study, the inverse association between MC and HPV detection was restricted to the coronal sulcus/glans penis [20]. A recently published longitudinal study conducted in the US among 477 male university students, uncircumcised men were also more likely to have HPV-positive glans and urine specimens than circumcised men, whereas circumcised men were more likely to be positive in the shaft/scrotum [34].

Taken together, results from these studies consistently show that the protective effect of MC may be restricted to infections occurring at the distal part of the penis, including the urethra, the coronal sulcus, and the glans. Since MC is limited to the removal of the foreskin, it seems unlikely that MC has any effect on infections occurring in the rest of the penis or the scrotum. Indeed the use in our study of an aggregate outcome that combined infections in more than one anatomical site greatly limited our ability to identify a true protective effect in specific anatomical subsites, possibly resulting in an overall underestimation of a MC protective effect.

It is interesting to note that this anatomical site-specific pattern of HPV infections is also observed for genital warts in at least two clinical studies [35,36]. One study of men attending an STD clinic in England found that circumcised men presented genital warts more often on the shaft of the penis and less often on the glans penis than uncircumcised men [35]. Similarly, in a study of heterosexual men attending an STD clinic in Washington, circumcised men presented genital warts more often on the penile shaft whereas uncircumcised men presented genital warts more often on the glans, corona, frenulum or meatus [36]. It is clear from these clinical studies that MC differentially reduces the risk of genital warts in the glans as compared to the rest of the genital area. As approximately 90% of genital warts are related to HPV 6/11 infections [37], these data are consistent with our finding that MC is associated with a significantly reduced prevalence of HPV 11.

It is unclear why MC would be associated with a reduced detection of non-oncogenic HPV types but not of oncogenic HPV types. One possible explanation is that MC infers a weak protective effect capable of preventing only less virulent HPV infections such as those caused by non-oncogenic types. Indeed, some studies provide evidence that non-oncogenic HPV types have a shorter persistence than oncogenic HPV types [7,38,39]. However, our own previous data from the *HIM Study* showed that the median time to clearance was not substantially different between non-oncogenic and oncogenic HPV types [40].

In this study, the distribution of the most common HPV types was not different by MC status. However, for less common types, such as HPV types 11, 40, 61, 71 and 81, we did find a lower prevalence in circumcised men than in uncircumcised men. Four studies have reported on the type-specific HPV distribution by MC status [20,32,34,41]. In a clinical trial of MC among young men in South Africa, HPV types 40, 42, 53, 70, 84, and CP6108 were found in the intention-to-treat analysis to be less frequently detected in circumcised men as compared to uncircumcised men when using urethral samples [32]. In another study of university males in Hawaii, the distribution of non-oncogenic HPV types in the coronal sulcus/glans penis did not vary by circumcision status, HPV 84 being the most common non-oncogenic type in both circumcised and uncircumcised men. However, the distribution of oncogenic HPV types did vary by MC status. Thus, while the most common oncogenic HPV types among circumcised men were types 16 and 39, those among uncircumcised men were types 66, 52, 53, and 73 [20]. Consistent with these two studies on type-specific prevalence, an RCT in Uganda found that type-specific incidence rates of HPV types 18 and 33 were lower in circumcised men than in uncircumcised men. Similarly, type-specific clearance rates for HPV types 39, 51, and 58 over a 24-month period were increased in circumcised men as compared to uncircumcised men when using samples from the coronal sulcus/glans penis [41]. Finally, in a longitudinal study of university males in Washington using samples from the penile shaft/scrotum, corona sulcus/glans, and urine, the incidence of the most common HPV types did not vary by circumcision status. The most common oncogenic HPV types at the 36-month cumulative incidence were 16, 18, and 51, and the most common non-oncogenic HPV types were 6, 53, and 66 in both circumcised and uncircumcised men [34].

The mechanism by which circumcision might protect against HPV infection remains unclear. Removal of the foreskin could minimize the chance of acquisition of new infections or could result in an increased clearance of preexisting infections [4,42]. Additional data on HPV acquisition and clearance according to MC status and anatomical site are necessary to better assess the role of MC in the natural history of HPV infections in men.

## Conclusions

In conclusion, this study shows no association between MC and an overall prevalence of genital HPV infections in men but MC is associated with a reduced prevalence of genital non-oncogenic HPV types. However, the lack of association with MC might be due to the analysis could not asses specific associations in the glans penis, the area expected to be most likely protected by removal of the foreskin.

## Abbreviations

HPV: Human papillomavirus; STI: Sexually transmitted infections; MC: Male circumcision; RCT: Randomized controlled trials; HIM Study: HPV in men study; CASI: Computer-Assisted Self-Interview; PRs: Prevalence ratios; CIs: Confidence intervals.

## Competing interests

G.A. My research unit is involved in vaccine trials organized by GlaxoSmithKline and Merck/Sanofi Pasteur MSD. Travel fund to conferences/symposia/meetings are occasionally granted by either GlaxoSmithKline, Sanofi Pasteur MSD or Qiagen. A.R.G. and L.L.V. are on the speakers’ bureau for Merck and are members of its advisory board. A.G.N. has received research funding from Merck & Co. X.C. Research Grants (GlaxoSmithKline, Merck Sharp & Dohme, Sanofi Pasteur MSD); Speakers Bureau (GlaxoSmithKline, Sanofi Pasteur MSD); Steering Committee (GlaxoSmithKline, Sanofi Pasteur MSD). F.X.B. Advisory Board (GlaxoSmithKline, Merck Sharp & Dohme, Sanofi Pasteur MSD); Speakers Bureau (GlaxoSmithKline); Research Grants (Merck Sharp & Dohme, Sanofi Pasteur MSD). X.C. and F.X.B. My research unit is involved in vaccine trials organized by GlaxoSmithKline and Merck/Sanofi Pasteur MSD. Travel fund to conferences/symposia/meetings and honorarium are occasionally granted by either GlaxoSmithKline, Merck, Sanofi Pasteur MSD, MTM or Qiagen. All other authors declare that they have no competing interests.

## Authors’ contributions

ARG, LLV, and EL were the principal investigators, conceived the study, wrote the protocols, assured funding, identified clinical investigators and study personnel, and made critical revision of the manuscript. GA was responsible for conduct analysis, interpretation of data, and drafting and revision of the manuscript. WF, and MRP made the statistical analyses, and made substantial comments to the manuscript. MA trained and supervised study clinical staff, and made substantial contributions to the manuscript. DS wrote the HPV assays protocols, performed the HPV DNA detection and genotyping of the samples validating the techniques with other referent HPV laboratories and made substantial comments to the manuscript. JS and MQ coordinated the field work in terms of study implementation and data collection, and made substantial comments to the manuscript. AGN, BL, XC, and FXB participated in the interpretation of data, and critical revision of the manuscript. All authors read and approved the final manuscript.

## Pre-publication history

The pre-publication history for this paper can be accessed here:

http://www.biomedcentral.com/1471-2334/13/18/prepub
